# Supporting Bereaved Family Members: A Qualitative Interview Study on the Experience of Bereavement Counselling by the Bereavement Network Lower Saxony (BNLS) in Germany for Parents Who Have Lost Children or Teenagers

**DOI:** 10.1177/00302228241263367

**Published:** 2024-06-22

**Authors:** Rebecca F. Kruse, Stephanie Stiel, Sven Schwabe

**Affiliations:** 1 Institute for General Practice and Palliative Care, 9177 Hannover Medical School, Hannover, Germany

**Keywords:** prevention of mental illness, surviving, grief, suicide, infant death

## Abstract

The “Trauer Netzwerk Niedersachsen” („Bereavement Network Lower Saxony“ (BNLS)) aims at supporting families after the loss of a child or teenager due to various causes. This study aims to describe the experiences of bereaved family members with the BNLS counsellors. 12 semi-structured interviews were conducted with parents who had received or were currently receiving BNLS counselling. The interviews revealed the vital role counselling played aiding individuals cope with their grief. Participants valued the bereavement support, which was often lacking in their personal support networks. Counselling assisted parents in returning to daily life and caring for loved ones. Discussing “death” and “dying” helped participants find peace with their loss. Our findings suggest that bereavement counselling should be considered an essential component of healthcare for family members dealing with the loss of a child. Additionally, there is need for awareness and publicity for both the BNLS and its bereavement counselling services.

## Background

The impact of a child’s or teenager’s death from sudden infant death, accidents, suicide, or life-limiting illnesses can have a tremendous effect on families, especially parents. Life-limiting diseases and death disrupt the family’s normal course of life and can also create emotional turmoil for the individual parent, and for parents as a couple (Lohan & Murphy, 2006, 2007; Oliver, 1999; Wheeler, 2001).

From the current literature we know, that losing a child may go ahead with numerous challenges. Problems can include (but are not limited to) taking care of surviving children and their grief, and adjustment to the new situation (Alvis et al., 2022). Bereaved families or individual members might start feeling isolated from other family members and/or friends (Bucaro et al., 2005; Price & Jones, 2015) or can experience a loss of identity (Horowitz, 2018). More individual challenges such as grief specific to the exact cause of death (e.g., guilt from suicide) might occur.

In addition to the above-mentioned challenges in every-day life, parents of deceased children are susceptible to a variety of negative psychological outcomes such as depression, complicated grief, and anxiety disorders along with social impacts such as difficulties to maintain and a lack of interest in social contacts, and a decreasing ability to perform effectively at work (Grande et al., 2015; Lobb et al., 2010). Families are confronted with the parents’ grief as well as with the remaining siblings’ grief which can present very differently, according to the child’s age, developmental stage, and cognitive abilities (Alvis et al., 2022; Lohan & Murphy, 2007).

Studies suggest that early intervention in parental grief can be helpful in preventing mental disorders, including depression and complicated grief (Aoun et al., 2015a; Jens C. ; Keyes et al., 2014; Lannen et al., 2008; Lobb et al., 2010; Nielsen et al., 2017; Shear et al., 2011; Thimm et al., 2020).

Interventions often include but are not limited to psychotherapy (with or without medication), support groups and (spiritual) counselling.

According to the “Complicated grief therapy” (CGT) two major focuses should be addressed: loss (e.g. imaginal revisiting) and restoration (e.g. personal goals and self-care) (Shear, 2010). This intervention also suggests using techniques known from cognitive-behavioral therapy (Shear et al., 2005).

Measurable positive effects of a bereavement intervention on sleep and cortisol secretion have been observed in previous research (Buckley et al., 2012). The abovementioned decrease the necessity of other health care services, thus providing a huge preventative factor (Saultz & Lochner, 2005; Shear et al., 2011). Research also indicates that preparation for the death of a loved one can also be beneficial to the psychological adjustment post-mortem (Barry et al., 2002; Maciejewski et al., 2007).

Bereavement counselling can offer parents and families support customized to align with their specific requirements, be it after the child’s passing or while caring and grieving for a child still alive but terminally ill. In Germany, bereavement counselling is offered by volunteers such as church organisations, hospice services or other organisations. We assume that the offers of these services differ from each other because there is no standard defined. Health care insurance providers in Germany do not finance these services systematically (Deutscher Hospiz- und PalliativVerband e.V, 2020; Familienportal Des Bundes, nd). In the context of bereavement care, health care insurance may cover psychotherapy expenses when deemed a necessary component for addressing grief. This could be the case if another disease according to the “international statistical classification of disease and related health problems” (short: ICD) is present (Betanet, nd).

One of these volunteer services is the “Trauer Netzwerk Niedersachsen” (engl. „Bereavement Network Lower Saxony” (BNLS)) which specifically offers bereavement counselling for family members in Lower Saxony, Germany, during the course of a life-limiting disease and after death. The BNLS focuses on supporting families coping with children afflicted by life-limiting diseases, as well as those navigating the challenging aftermath of deaths stemming from various causes, including accidents and suicides. The support families received varied according to the individual needs and backgrounds of the child´s passing. While some families utilised the services of the BNLS mainly for their own grief or guilt in a case of suicide, others appreciated the support they were provided for taking care of surviving siblings.

The BNLS was launched in January 2021 as part of the Network for the Care of seriously ill children and adolescents (“Netzwerk für die Versorgung schwerkranker Kinder und Jugendlicher e.V.”) (Betreuungsnetz, nd). Families using these services are not charged as the BNLS runs on donations and external funding. All of the bereavement care providers working in the BNLS have completed the major foundational qualification course by the German federal association for bereavement care. The bereavement care providers working in the BNLS have individual backgrounds such as hospice services, social work, psychology or are members of psychosocial teams or nurses (Betke et al., 2024). Qualifications of the bereavement care providers are ensured according to the official requirements of the German federal association for bereavement care e.V. (Bundesverband Trauerbegleitung (BVT)). Requirements include the following components: completed apprenticeship or university degree and 200 teaching units of 45 minutes each.

### Study Aim

This study aims to describe how bereaved family members experience the support by bereavement counsellors working for the BNLS.

## Methods

### Design

To address the main research aim, a qualitative interview study with parents who receive(d) bereavement counselling by counsellors within the BNLS was conducted.

### Study Material

To determine which topics are important for the exploration of parents’ experiences to include in our semi-structured interview guide, a workshop was held with bereavement counsellors, members of the BNLS, and the research team. In accordance with the topics which arose, a semi-structured interview guide was developed, assessed in terms of comprehensibility, and consented. The interview guide consists of 30 open questions in four categories: initial contact, experience with the bereavement counselling, communication modalities, and reflection.

“Initial contact” includes questions such as why bereavement support with the BNLS was started, who introduced the BNLS to our participants and what kind of hopes and fears were associated regarding bereavement support.

“Experience with the bereavement counselling” includes questions regarding topics, emotions, influence, and effects the BNLS had on our participants.

In the section “communication modalities” we asked our participants how they were in contact with their bereavement care provider and/or the BNLS. Lastly, the section “reflections” includes questions about what effects the BNLS and bereavement support had on our participants as well as whether they would partake in bereavement support again or recommend support for others.

### Sampling and Recruitment

Firstly, counsellors were contacted via email with a recruitment letter by the research team (RFK, SvS, SSt). Secondly, counsellors approached their families they had supported or were currently supporting if they would be willing to participate in the research project.

Following the recruitment letter distribution by the bereavement counsellors, families either contacted the research team directly or through their bereavement counsellors. Further documents were given to potential participants, including an information sheet, a socio-demographic questionnaire, and a consent form to be signed and sent back to the research team prior to the interview. Dates with participating parents for interviews were scheduled individually via phone or email.

## Data Collection

12 semi-structured interviews were conducted by RFK with one parent each who had lost a child and had been or was being counselled by members of the BNLS (see Table 1). The interviews took place between July 2022 and April 2023. All interviews were held either online by the video conference platform BigBlueButton or by phone, and were audio recorded on an Olympus recorder. Due to technical malfunctions one interview was recorded on the interviewer’s iPad.

**Table 1.** Sociodemographic Data of Participants of the Semi Structured Interviews (n = 12).

Characteristics		*n* (%)
Sex	Female	11 (92%)
	Male	1 (8%)
Age (years)	Mean	43.18 (median 41)
	Range	22 – 62
Marital status	Married	10 (83%)
	Mean time married (years)	15.4
	Relationship	1 (8%)
	Divorced	1 (8%)
Religion	Protestant	11 (92%)
	Christian	1 (8%)
Educational degree	High school	5 (42%)
	Secondary school	7 (58%)
Professional degree	Apprenticeship	8 (67%)
	Master	1 (8%)
	Master/PhD/Doctorate	2 (17%)
	Diploma	1 (8%)
Employment	Full-time	3 (25%)
	Part-time	7 (58%)
	Care-work (“Stay-at-home” parent)	1 (8%)
	No employment	1 (8%)
Profession	Domestic service worker	2 (17%)
	Editorial leadership in Content Marketing	1 (8%)
	Teacher	2 (17%)
	Medical Assistant	1 (8%)
	Technical assistant	1 (8%)
	Nurse	1 (8%)
	Office clerk	1 (8%)
	Team Leadership	1 (8%)
	Chemistry laboratory technician	1 (8%)
	Nurse´s assistance	1 (8%)
Residency	Rural	7 (58)
	Urban	5 (42%)
Number of children	Mean	2.5
	Range	1 - 4
Cause of death of child	Accident	3 (25%)
	Suicide	1 (8%)
	Brain tumor	3 (25%)
	Genetic disorder	1 (8%)
	Undefined	3 (25%)
	Stillbirth	1 (8%)
Age of child at death (years)	Mean	8.23 (median 5)
	Range	0 (stillbirth) – 24
Duration of bereavement counselling with BNLS (month)	Mean	15.18
	Range	12 – 24 (partially ongoing, median 16)
Ongoing bereavement counselling with BNLS after child`s death	Yes	10
	No	2

### Data Analysis

According to Mayring’s (Mey & Mruck, 2010) qualitative content analysis, the acquired data was coded using MAXQDA (i.e. qualitative data analytical software). Codes were developed by RFK and reviewed and consulted on by SSt and SvS. Many categories were coded deductively based on the interview guide, whilst other categories and codes arose in the further coding process. The codes were structured into main categories focusing on the experiences parents had while undergoing bereavement counselling by the BNLS. This process created a code tree (see Table 2) including major code groups with subcategories. Key examples were collected and annotated with definitions to establish a clear framework of each code and sub coding.

**Table 2.** Code Tree.

1. Initial contact	Own research for bereavement counselling
	Obstacle for contact
	Course of first meeting
	Timely relationship to death of child
	Contact initiator
	Contact to bereavement counsellor already pre-existing before commencing bereavement counselling with BNLS
2. Factors influencing the decision to commence bereavement counselling	Upkeeping of memories of deceased child
	Handling grief
	Finding normality
	No expectations
	Help
	Gaining of neutral and experienced conversation partner
	Handling of remaining sibling
	High motivation
	Hesitations to commence
3. Experience of and within bereavement counselling	Effects of bereavement counselling
	Meaning of bereavement counselling
	Emotional support
	Content of bereavement counselling
	Participants of bereavement counselling
	Perception of bereavement counsellor
	Emotions during bereavement counselling
	Culture/Religion
4. Organisation of bereavement counselling	Contact to bereavement counsellor
5. Contact to BNLS	No contact to BNLS
6. Further offers of bereavement counselling (outside of the BNLS)	Psychological guidance
	Palliative care facilities
	Sibling-, Children- and/or adolescent groups
	Hospice care
	Church
	Peer groups
	No further offers
7. Reflection	Currently ongoing counselling
	Recommendation
	Situations and Conditions for recommendation
	Potential for improvement, praise, and criticism

## Results

The 12 semi-structured interviews lasted between 20 and 66 minutes (median: 28 minutes). The number of female participants was 11, and the participants’ average age was 43.2 years (range 22–62 years, median: 41 years). Each interview was held with one individual parent of a deceased child. On average, bereavement counselling was accessed for 15.2 months (median: 16 months). Deceased children ranged ages from 0 (stillborn) to 24 years (median: 5 years). Seven major categories regarding different aspects of the perception of bereavement counselling by the BNLS arose with each 1-9 subcategories (see Table 2).

### Initial Contact

Establishment of contact to the bereavement counsellors varied greatly. More (6 of 12) parents encountered their bereavement counsellor via acquaintances. Parents were introduced to the BNLS or bereavement counsellors by medical staff such as psychologist or paediatricians. These healthcare professionals recognised the parent´s grief as too complex for their respective field of expertise or recognized the parent´s need for bereavement counselling.

Some (2 of 12) parents had known their bereavement counsellor through other institutions which then also eased into the decision to commence bereavement counselling by the BNLS. Others were introduced to the BNLS or their bereavement counsellors by friends. Only a few (2 of 12) parents looked for guidance and help with and through their grief by themselves and thus, found the BNLS or bereavement counselling online.

“Well, I didn´t really become aware of it but I looked for someone here in my vicinity or my surroundings who could support me in my grief. That´s when I came across my bereavement counsellor and I didn’t even know which network she actually worked with, but she was nearby, and she sounded quite positive on her website. So I though, I’d approach her, maybe she can help you.” (Mother of a deceased child)

Most (10 of 12) parents reported that they met their bereavement counsellor after their child’s death. Temporal proximity to the children’s death varied between less than 24 hrs and 6 years.

As reported by the interviewees, the very first contact to the bereavement counsellor was by phone or via email to arrange a personal meeting as soon as possible. These personal meetings varied in their nature, depending on the temporal proximity to the death, and on the environment in which these took place. Some parents chose to visit the bereavement counsellor in their offices, others preferred to be visited in their own homes.

“And she came to visit us, uh, paid us a visit. Just dropped by for a coffee, visited [person] and also did something with [person].” (Father of a deceased child)

### Factors Influencing the Decision to Commence Bereavement Counselling

Motivation to commence bereavement counselling can be classified into major groups: (a) parents wish to keep the memory of their child up, (b) the desire to understand and learn how to deal with grief, (c) receiving help and finding a new sense of “normality”, (d) having a neutral but experienced conversation partner, and (e) learning how to handle remaining siblings.

Many parents (9 of 12) found themselves in a position in which they were trying to comprehend the situation while friends and family who had known the child were also grieving. Hence, parents could not help the grieving family sufficiently. Interviewees reported that the neutrality and specialisation of the bereavement counsellors on grief over a child was a favourable factor for the bereavement counselling.

“We noticed that it’s beneficial for us to talk to people who are outside of our family. They are, so to speak, “neutral” and, not biased when it comes to our feelings or how we handle the situation. It’s always a bit tricky with the family, because they are also affected themselves. That’s why we had the hope that there are people who can provide us with support, guidance, or maybe even have an idea about how to handle the whole situation differently.” (Mother of a deceased child)

In addition to the above mentioned, parents with more children were often confronted with the deceased child’s siblings‘ questions.

“…he turned five and said “Mom, how old was my sister?” and then I said “She turned 5” and then he said “Mom, but I will be older, right?” “yes”, I say, “you will be older. You are healthy, everything is... And we take care of you” And then he says “Yes, but Mom, why didn’t you take care of her?” And those are the situations when I wish… oh, there… well, I just couldn’t answer. I just left it at that and, uhm, talked to [my bereavement counsellor] about it and yes… This is my wish for this support… that I can handle things better with [the siblings], that I can sometimes handle myself better. It really isn’t easy.” (Mother of a deceased child)

Four of ten parents had the opportunity to prepare for the child’s death, since most of the children had suffered a terminal illness. Six of ten parents’ children had died without any previously known diseases and therefore no time to prepare. For both groups learning how to deal with their grief was equally important and played a role in the decision for bereavement counselling.

“I, um hoped that I could somehow get through this situation. So let’s say after the death, you didn´t really know what the future held, I would say. Uhm, yes, the hope was to learn to live with it and not be so desperate forever and to deal with the whole problematic… issue, I would say. So the support was important to me, that you could get through it.” (Mother of a deceased child)

The fear of forgetting their child motivated one parent to commence bereavement counselling.

“Somehow, you don’t take that time in your everyday life. And that was perhaps an unspoken wish that has been fulfilled.” (Mother of a deceased child)

Not every parent went into contact with a bereavement counsellor or started bereavement counselling with specific expectations. Two parents specifically said they could not have any expectations, as it is not possible to have expectations for something you have never experienced before.

“I can’t really say because you go into something you haven’t experienced without expectations. Or I don’t enter into it that way. I can’t expect anything if I haven’t experienced it. And uhm… Besides, one is so shaken after the death of a child that you don’t have any expectations for such things. It’s just… In the beginning, the first few weeks you’re like numb and lifeless, and you’re just trying to survive, so you have no expectations.” (Mother of a deceased child)

While mostly positively influencing factors to commence bereavement counselling were mentioned, five parents also reported reservations.

“Fear, of course that everything will get worse or, well, that grief won’t end.” (Mother of a deceased child)

### Experiences of and within Bereavement Counselling

Conversations were the core component of all the interviewees’ bereavement counselling.

“She’s really good at listening. We could talk a lot with her.” (Mother of a deceased child)

Half of the interviewed parents reported that they had sole participation in bereavement counselling. The other half reported other family members, such as partners and remaining children or grandparents of the deceased child participating in bereavement support at least once.

“My mother, so [my child’s] grandmother joined me for one conversation but otherwise [the bereavement counsellor] and I were always alone.” (Mother of a deceased child)

### Significance

The meaning of the bereavement counselling can be summarized into five categories: (a) understanding for the situation, (b) an objective conversation partner, (c) organisational support, (d) accessibility at any time and (e) emotional guidance.

Three parents reported they did not experience long-term sympathy for their situation. Parents reported their friends and acquaintances found it difficult to understand them as they did not understand that grief was not a linear process and parents would present a positive attitude at one time but later on show a subjective “decline”. Bereavement counsellors understood the non-linear process and were able to be a pillar for parents in this situation.

“Because, uhm, it’s like your immediate circle can’t really understand. So, for those who haven’t experienced it, they simply can’t understand, that you can feel different at different times, that you can feel good some days but then on others you feel bad again, even if you felt good before. It’s really a kind of “up and down”. And that’s why I found it increasingly difficult to talk to people around me about how I am doing because I always had a feeling I wouldn’t be understood. And that’s what I found good, that I could talk to my bereavement care provider about this.” (Mother of a deceased child)

Parents also found it difficult to talk about the situation with family and friends since this group of people mostly knew the deceased child and were also grieving the death of the child. Bereavement counsellors were appreciated as people the parents felt comfortable to talk to about death and their experiences.

“Yes, I definitely have someone there who listens, with whom I can talk about the death.” (Mother of a deceased child)

Bereavement counsellors offered the parents organisational help and were available at any time if the parents felt the urge to talk or needed guidance through difficult situations. This meant a lot to the interviewees.

“But of course, I also have the possibility to text her or to call her if there’s an emergency. There are just some days when you think “Wow, this is getting dangerous, I can’t get out of this myself” or “I don’t know how to handle this” but she is always reachable in those moments.”(Mother of a deceased child)

The bereavement counsellors portrayed emotional support by helping the parent understand their own feelings while also offering guidance and comfort.

“She was, uhm, yes, a help… She’s an anchor, support. I mean, it’s like… you just have to see after all that happened, that you can get back into life. And that’s… most of it you have to do by yourself. But it’s still, when you feel like you’re falling, having a hand that you can reach out to again. Getting up and everything, I have to do it on my own, but it’s like a symbolic hand that can assist you.” (Mother of a deceased child)

#### Content

All interviewees mainly reported grief, life after loss, and death as core components of their bereavement counselling.

“We spoke a lot about death, yes. Dying… how to continue…” (Mother of a deceased child)

Another core component of bereavement counselling was not to forget the deceased child and to include the memory of the child in family life after death, the approach to this differed depending on the individual circumstances.

“How, uhm, how we can incorporate our daughter, so she’s not forgotten. Something that, as parents, we definitely don’t want.” (Mother of a deceased child)

A major issue of bereavement counselling for parents was dealing with other grieving family members and friends.

“Well... uhm, friends and families are simply overwhelmed. It’s not Granny or Grandpa who have died, it is my child and, uhm, that’s a different level of understanding. And that’s also a feeling, or maybe not a feeling but that I am a burden. So, when I contact a friend or my mother in such a phase and I am really not doing well with grief, I just always feel like I am being a burden for that person, because death of a child is really not easy to endure.” (Mother of a deceased child)

Other topics that arose during bereavement counselling were more individual, such as guilt after suicide.

“I mean, there’s a lot to it, it’s practically, it’s suicide. It’s feelings of guilt, guilt. “Why couldn’t I…”, so the question of “why? Why couldn’t I help?” ” (Mother of a deceased child)

#### Influence

Grief, death and dying as content of bereavement counselling positively influenced parents in how they dealt with these topics in their everyday life.

“Definitely effective! [...], I notice an extreme difference. Um, because it hasn’t been long for us... I'm already much further along in many topics than people who haven’t had such contact. Um, yes, I can tell that with them, it’s sometimes been 10 years or more, and they really... nothing has been processed, you can feel the difference.” (Mother of a deceased child)

Interviewees reported effects on their everyday life which can overall be summarized as more self-awareness about emotional and mental needs which differ in execution. One parent started daily “gratitude diaries” and nearly all parents were more mindful of their capacities and made sure to respect their individual boundaries to maintain emotional and mental sanity.

“[…] I have also started something. I began keeping a gratitude journal where I write down what I’m thankful for every day. We initiated this because I was struggling to see the positive aspects of my day, and I wanted to make an effort to recognize the beauty in it. This practice has been helpful, especially during times when it's challenging to find things to be grateful for.” (Mother of a deceased child)

One interviewee was so positively influenced by their experience of having bereavement counselling that she decided to become one herself.

“Yes, it definitely made me stronger, I would say. Uhm, yes and I am now thankful for all of life and for people, for my support which paved… I am now also offering support during dying and death. I just finished my course. Yes, that’s what you can say.” (Mother of a deceased child)

Many (7 of 12) parents reported bereavement counselling changing their perspective on the future.

“Yes, I really believe it helped me to get out of certain negative mindsets and to allow a different perspective.” (Mother of a deceased child)

#### Perception of Bereavement Counsellor

Every interviewee spoke positively of the individual bereavement counsellor they had been or were in contact with. They were described as sympathetic, considerate, and attentive.

“Very open, empathetic, and very, yes helpful.” (Mother of a deceased child)

Half of the parents reported their relationship with their bereavement counsellor resembled a friendship.

“The relationship to [my bereavement counsellor] I think I already said… It feels like she is a friend because, I mean I can only talk for her, I don’t know how others work but we engage in conversation, she lets me know what’s going on in her life. So that… My trust in her is higher and uhm I don’t have the feeling that I... I mean she can clearly say if something is going too far. She could say “hey, that’s too much” but, uhm, she’s very professional. “Professional friends” sounds good, but a very friendly relationship. Definitely.” (Mother of a deceased child)

In addition, bereavement counsellors were perceived as flexible regarding location and time of meetings.

“Something else I experienced very positively, that uhm, we could occasionally meet at the graveyard. That the bereavement care provider... that they just said “Yes, of course, we’ll meet at the graveyard and I’ll come there” and uhm that this was possible with the kids, too, if they wanted to. Because, well for me the graveyard is a very important place. For some people it might not be, or they don’t have a connection, but the fact that it worked in a location-independent way or at the place where you felt comfortable doing it, I found that very nice.” (Mother of a deceased child)

Bereavement counsellors also demonstrated high level of commitment to the parents. This could be a message or postcard for anniversaries or transporting the parents to the graveyard.

“And we still have contact to the lady who visited us. She occasionally reaches out to us. We also get, at least that’s how it was until now on birthdays or the anniversary of the passing, we received a little card or a text message […] she wrote to us personally. Personal notes are nice because you know you’re not forgotten.” (Father of a deceased child)

#### Emotions during Bereavement Counselling

Bereavement counselling was an emotional undertaking for the parents interviewed. The idea of support itself triggered doubts in five parents.

“When I had my first conversation, I was thinking, “Is this really how it should be?” Usually, I’m the type of person who deals with things on my own.” (Mother of a deceased child)

Emotions that became evident through the conducted interviews varied extensively. This depended on the background of the child’s death, the course of disease, the individual situation and the length of counselling which was given by the BNLS.

Three parents reported grief, dismay and nostalgia.

“I’m still in my grief, and perhaps I haven’t really come to terms with the fact that my son is no longer here. Or that I can’t let go of him, and that’s why I can’t understand why my faith allows something like this to happen to me. That I must endure such a heavy fate.” (Mother of a deceased child)

More positive and hopeful emotions included gratitude, security, recognition, joy, hope and relief.

#### Religion

Overall religion was a minor component of bereavement counselling to few interviewees. Bereavement counsellors were open to include the parents’ religious beliefs in their support but were generally described as neutral and reserved.

“Open, neutral. […] it wasn’t discussed. Although I did mention the baptism of the children, it wasn’t explored further. It was very neutral and restrained.” (Mother of a deceased child)

### Organisation of Bereavement Counselling

In only a few cases the BNLS was part of the communication process by establishing contact to the bereavement counsellor. After this each parent and bereavement counsellor pair maintained their contact directly.

While the meetings were conducted in person, organisational aspects such as when and where the next meeting would take place were almost exclusively handled online by means of messenger systems such as What’s App, emails, or phone calls.

“So, we personally coordinated the appointments […] when we had our appointments. I could have also reached out personally, whether by phone, email, or mobile number. It was entirely open for me to decide when and how I wanted to contact her in between appointments […] as needed.” (Mother of a deceased child)

Frequencies of meetings ranged between daily to once a month or as needed. This was individually planned as needed and varied for each parent and their counsellor according to their need at the given time.

“Later on, we did individual appointments as needed with my bereavement counsellor or we just spoke.” (Mother of a deceased child)

Some bereavement counsellors had their own offices in which they held their sessions with parents, while others visited the parents in their homes or in other locations, such as the graveyard. The decision was heavily influenced by the parent and their wish and need at the given time.

“She came to my home and I really liked that because that’s where I feel good and I didn’t have to go to another location. So it was less work for me, I just had to open the door.” (Mother of a deceased child)

### Contact to the BNLS

No parent reported having been in contact with the BNLS as a network, on the contrary, four interviewees specifically said they had no contact with the BNLS, just with the specific counsellor of the BNLS.

“I didn’t have contact with the BNLS at all, contact with me was all through [my bereavement counsellor]” (Mother of a deceased child)

### Further Support Outside of the BNLS

In addition to the bereavement counselling by the BNLS, parents also utilized other services which included church, psychologist, palliative care facilities, hospice care facilities, peer groups, and groups for children and adolescents (for siblings). In seven cases, these services established the first contact to the BNLS or the individual bereavement counsellor. By far, peer groups were the most mentioned external service parents took part in. Officially organised peer groups by services such as the BNLS were often discontinued by the parents since they did not feel the groups were meeting their needs or were not appropriate for their situation.

“I had that right after the death. However, it didn’t help me, or those were parents who had lost only one child, and I still had two at home... And that made me feel rather guilty for still having children while they didn’t, so I stopped attending.” (Mother of a deceased child)

On the contrary, peer groups organised unofficially by parents with similar circumstances of death were often continued and supported parents in a way they felt appropriate.

“We don’t call ourselves a grief group, but rather “Shared Parents with Shared Fate.” This allows us to understand, what those who have gone through the first year of grieving have experienced. There’s also a new family that recently lost their child in a fatal accident in February, whom I haven’t met yet. However, they might handle the situation better than someone who has never experienced this. I believe that their presence could be supportive. These families all live within a 30/40-km radius, which I think makes it easier than constantly driving to any group where there’s a family that has lost a baby. They can’t relate to what I’ve been through.” (Mother of a deceased child)

### Reflection

At the time of the conducted interviews, six parents were still being counselled by their bereavement counsellor.

“It’s not finished yet, it’s still ongoing and I think we will still spend some hours with each other.” (Mother of a deceased child)

One interviewee specifically mentioned they wished for prolonged counselling.

“For me, there is nothing negative. I miss the time, I would like to have had more time.” (Mother of a deceased child)

Overall, parents interviewed would recommend bereavement counselling by the BNLS, and they would also wish to be counselled again should there be any need for it.

“I would do it again, anytime and would recommend it anytime, too.” (Mother of a deceased child)

Ideas of improvements include more differentiated offers according to individual circumstances and age of the deceased child.

“I also sometimes struggle within the grief network, because, well, it’s a different kind of grief whether you lose a child at twenty-four or if the child was only six years old. But I don't really want to say something like that now, because, in the end, I don't have much... wait…, experience with parents who have lost very young children.” (Mother of a deceased child)

Parents also mentioned that prominence of the bereavement counselling by the BNLS should be improved as many people had not heard of bereavement counselling.

“More popularity. Well, it’s still kind of a secret that, thankfully, has reached me. When I told many relatives, friends, and acquaintances about it during the mentioned fundraising marathon, they didn’t really know much about it. […] I would really like it if they could gain a larger lobby.” (Mother of a deceased child)

Financial incorporation of health insurances to support families more comprehensively during grief was also mentioned.

“But I think that health insurance should actually be obliged, because the risk that one, after severe grief, one slides into depression, that you get the disease of depression, is quite high I believe.” (Mother of a deceased child)

## Discussion

Conducted interviews with bereaved parents having undergone bereavement counselling with the BNLS highlight the following topics: (1) Initial Contact, (2) Factors influencing the decision to commence bereavement counselling, (3) Experience of and within bereavement counselling, (4) Organisation of bereavement counselling, (5) Contact to the BNLS, (6), Further offers of bereavement counselling (outside of the BNLS), and (7) Reflection.

This study highlights the importance of a holistic approach to grief following the death of a child or after receiving a diagnosis of a life-limiting illness. The individuality of bereavement counselling was highly beneficial to all participants on a multitude of levels, which aligns with previous studies (Butler et al., 2014; Donovan et al., 2014; Garstang et al., 2014; Gijzen et al., 2016; October et al., 2018).

In 2016 Gijzen et al. investigated how parents perceived support offered after the death of a child and came to the conclusion that parents valued health care workers maintaining contact six to twelve months following the death of a child, as it allows for periodic assessments of whether the family requires additional care or support. The same study highlights a lack of support by non-professionals, such as family. Especially the latter, which has also been observed in other studies (Bogetz et al., 2021; Levetown, 2008) aligns with our finding of parents not feeling adequately supported and understood by their friends and families and health care professionals. It is visible that parents found it difficult to approach their social network of friends and family due to their own emotional involvement regarding the death of the child. Parents longed for someone they could talk to, without having the feeling their conversation partner was being burdened by them expressing their thoughts. This also resonates with previous research, which suggests social networks are a necessary component of support but can still be inadequate in aiding navigating grief after child-loss. A combination of formal and informal assistance is part of bereavement care since they address different requirements (Donovan et al., 2014; Müller & Klaschik, 2001).

The combination of formal and informal support is a unique characteristic of the BNLS. Besides the mandatory major foundational qualification course by the German Federal Association for Bereavement Care, bereavement counsellors in this network typically had backgrounds in medical or psychological fields (amongst others), representing the formal aspect of the support system (Betke et al., 2024). Concurrently, the relationships the parents build with their bereavement counsellors tend to evolve into a more informal dynamic, resembling a friendship. Other facilities, such as church, psychologist, palliative care facilities, hospice care facilities, peer groups, and groups for children and adolescents (for siblings) all represent individual aspects of both formal and informal assistance, but their spectrum does not compare to what the BNLS offers. Besides the combination of the previously described formal and informal aspects the BNLS offers both on an individualized level. This individual approach was positively pointed out by our participants.

Several studies have examined the effect of interventions for parents and/or caregivers in the context of palliative care to assess if it decreases the incidence of complicated and severe grief or other grief-associated diseases (Aoun et al., 2015bAoun, Grande, et al., 2015; Grande et al., 2015; Northouse et al., 2010; Toye et al., 2016). Despite the different types of intervention most studies concluded a positive outcome for the patients and/or the caregivers. Our study did not specifically investigate the temporal aspects of bereavement counselling utilization, nevertheless, it is noteworthy that many participants engaged in frequent utilization of bereavement counselling in the initial phase of grief. A discernible decrease in the necessity of support sessions emerged after this intense initial phase. This observation corresponds to a meta-analysis which found a strong link between the time and effort invested into the intervention and the outcomes being more favourable (Northouse et al., 2010).

The emotional nature of research with bereaved families and the difficulties this portrays in procurement have been observed in other studies (Dyregrov, 2004; Stroebe et al., 2003). In alignment with a study conducted by Steele et al., (2013), parents who participated in this study often expressed gratitude by contributing towards further publicity of the BNLS. They felt that the support they had received was something they hoped would reach more families and parents (or any other individual struggling with grief).

Our participants highlighted several favourable attributes within the BNLS that distinguish it from alternative bereavement support approaches. On the one hand, the already discussed integration of both formal and informal components was a noteworthy factor. On the other hand, participants emphasized the significance of flexibility concerning aspects such as time, discussion topics, meeting locations, and session frequency. Small gestures, such as cards for anniversaries had tremendous impacts on our participants and were fondly remembered. This effect had already been observed when it came to health care professionals who had been in close contact with families preceding a child’s death (Levetown, 2008).

### Strength and Limitations

Interview partners were approached by the BNLS and the bereavement counsellors. Some bereavement counsellors did not approach their supported families with this request to avoid anticipated burden caused by study invitation and participation. Hence, a positive selection bias among the participants cannot be excluded. However, families initially excluded due to their heightened emotional involvement demonstrated a capacity to participate in later rounds of procurement.

The above-mentioned method of acquisition does not allow insight into non-responder characteristics. During our recruitment, we could have had access to around 50 families who had been or were being supported by the BNLS. Given the qualitative nature of the study and a small sample size, the 12 participants represent roughly 25% of the supported families from the BLNS.

Both ongoing and concluded bereavement supported parents were included in our cross-sectional study. Parents still undergoing bereavement counselling might have had other perspectives in retrospect if interviewed once their support was concluded and vice versa.

While the qualitative nature of this study provides detailed insight to the parent´s perspective, a quantitative study should be conducted to offer additional data to complement the findings.

We have a gender bias in our sample because all but one participant were female.

The coding process was conducted by RFK. To mitigate potential sources of error, two separate reviewers (SvS and SSt) checked the reliability and validity of the coding process. The primary source of data acquisition stemmed from interviews. Relying exclusively on data from interviews presents several limitations, such as a participant’s bias, dependency on memory and difficulties to generalize. This was mitigated by the use of a semi-structured interview guide which was used for every interview.

Since the BNLS does not differentiate who they can and will support a broad range of age of the deceased and the interview partners as well as many different causes of death and socio-economic backgrounds could be included in our research. This allows insight into issues that nearly all families endure during the course of a life-limiting illness, as well as during their grief, irrespective of the cause of death, pathway of grief and other contributing factors. Additionally, the timely distance to the death of a child varied greatly for our interviewees. This also contributes to the above-mentioned, while also giving insight into the progression of grief for parents.

## Conclusion

Bereavement counselling by the BNLS is a positive example of holistic bereavement care and contributes meaningfully to bereavement care for individuals in Lower Saxony, Germany.

In terms of bereavement care, parents expressed a desire to increase advocacy efforts and enhance insurance inclusion and coverage to bolster visibility and reach of the BNLS, thus enabling a larger section of families and/or individuals to access support services offered by the BNLS.

In conclusion it can be said that the BNLS effected grieving families positively and should be further utilised to aid any individual dealing with grief, especially families who have lost a child or teenager. Funding should be made available to offer more differentiated services tailored to a broader selection of backgrounds and to facilitate visibility.

Further research should investigate the course grief and quality of life in parents with bereavement support in a longitudinal design and use a control group design to test for effects of bereavement support in a larger population.

## Data Availability

The data used during the present study is available from the corresponding author upon reasonable request. It will be stored according to research guidelines.*
